# Right Hepatic Artery Pseudoaneurysm Post-laparoscopic Cholecystectomy: A Case Report of Endovascular Stent-Graft Management

**DOI:** 10.7759/cureus.57127

**Published:** 2024-03-28

**Authors:** Sohaib Ahmed, Rares Filep, Ahsan Mushtaq, Ovidiu Budisca

**Affiliations:** 1 Surgery - I, County Emergency Clinical Hospital of Târgu Mureș, Targu Mures, ROU; 2 Interventional Radiology, County Emergency Clinical Hospital of Târgu Mureș, Targu Mures, ROU

**Keywords:** graft, stent, complications, laparoscopic cholecystectomy, pseudoaneurysm

## Abstract

Gallstone-related diseases like cholelithiasis contribute significantly to global morbidity and mortality. Laparoscopic cholecystectomy (LC) is the gold standard for gallbladder removal but is associated with rare but severe complications, including hepatic artery pseudoaneurysms (PAs). A 72-year-old female presented with acute abdominal pain and upper gastrointestinal bleeding following a recent LC. Laboratory studies confirmed anemia with a hemoglobin level of 10 g/dL. Liver function tests were deranged, showing elevated alanine aminotransferase (ALT) at 209 U/L, aspartate aminotransferase (AST) at 472 U/L, total bilirubin levels at 3.29 mg/dL, and direct bilirubin levels at 2.7 mg/dL. A contrast-enhanced computed tomography scan revealed a PA adjacent to the right hepatic artery. Given the strong suspicion of a hepatic PA as the source of her symptoms, an endovascular stent was placed by an interventional radiologist. Post-procedure, the patient showed a favorable clinical course with cessation of symptoms and was discharged after eight days. This case emphasizes the importance of early identification of hepatic artery PAs following LC, a potentially life-threatening complication. It also suggests that endovascular stent placement can be an effective alternative to traditional transarterial embolization for managing these PAs. Additional research is needed to evaluate the long-term effectiveness and safety of these two methods in comparison.

## Introduction

In the United States and around the world, gallstone-related diseases like cholelithiasis and cholecystitis significantly contribute to morbidity and mortality rates, placing a substantial burden on healthcare resources [1]. Over 20 million people in the United States have been diagnosed with gallbladder disease through ultrasonographic evaluations [2]. Globally, the incidence of cholelithiasis varies between 10% and 15% among the general population, although this rate can differ based on geographical factors. Among those affected by gallstones, approximately 20% to 40% are likely to experience complications, which manifest annually at a rate of 1% to 3% [3-5].

Despite a significant decline in gallstone-related mortality rates from 1979 to 2004, with a 56% decrease in cases where gallstones were identified as the primary etiological factor and a 71% decrease in cases where they were a contributory factor, there were still 2,155 documented fatalities in 2004 attributed to gallstones as either a primary or secondary cause [6].

Data from the 2006 National Survey of Ambulatory Surgery, administered by the Centers for Disease Control and Prevention's National Center for Health Statistics [7], indicated that gallstone diseases culminated in 503,000 laparoscopic cholecystectomies (LCs), a procedure that is generally considered the gold standard for gallbladder excision, given their benefits such as reduced post-operative discomfort, quicker recovery, and decreased wound-related complications [7-9]. Nonetheless, despite its widespread acceptance and low associated risk, particularly when executed by proficient surgical teams, LC is not entirely without potential complications as the overall rate of severe complications associated with LC consistently surpasses that observed in open cholecystectomy procedures [10,11]. Among the less common but significant post-operative complications is the emergence of pseudoaneurysms (PAs), most frequently found in the hepatic artery and, less commonly, the cystic artery [12,13]. These PAs can manifest as post-operative hemobilia within weeks or, in more severe cases, spontaneous rupture into the peritoneal cavity. Upon diagnosis, favored treatment approaches include percutaneous catheter embolization, endovascular stent placement, or, in rare instances, surgical ligation of the affected artery [14,15].

## Case presentation

We report a case of a 72-year-old female who presented with severe abdominal pain and mild upper gastrointestinal (GI) bleeding manifesting as melena. She had a complex past medical history including a recent LC, multiple episodes of mild upper GI bleeding, and hiatal hernia repair.

Clinical history timeline

First Presentation (Initial Encounter)

The patient initially arrived with symptoms indicative of acute cholecystitis, including severe abdominal pain and fever. Clinical and paraclinical investigations confirmed the diagnosis. She underwent an emergency laparoscopic cholecystectomy. During the procedure, acute calculous flegmonous cholecystitis was identified and treated, and a hiatal hernia was also noted but left for later correction. She was discharged four days following the surgery.

Second Presentation (Two Months Later)

The patient was readmitted to the gastroenterology department with suspected obstructive jaundice and multiple episodes of upper GI bleeding of unknown origin. However, eco-endoscopic examination later ruled out obstructive jaundice. She received appropriate treatment and was discharged after two days.

Third Presentation (One Month After the Second Presentation)

The patient returned for a planned laparoscopic correction of her hiatal hernia. She was operated laparoscopically; the hernia was corrected through recalibration of the hiatus and a Nissen fundoplication for a large sliding hiatal hernia. She was discharged after a week's stay.

Current Presentation

The patient was admitted from the emergency department with acute, intense abdominal pain and signs of upper GI bleeding externalized through melena.

Initial investigations

Laboratory investigations at the time of admission revealed anemia and deranged liver function tests, as shown in Table 1. An initial abdominal CT scan showed a dilated common bile duct (CBD) measuring 12 mm, splenomegaly, and the absence of a gallbladder, consistent with her history of cholecystectomy.

**Table 1:** Laboratory Investigations at Admission

Laboratory Test	Result	Reference Values	Unit
Hemoglobin	10	12.1 – 15.1	g/dL
Alanine Aminotransferase (ALT)	209	10 – 130	U/L
Aspartate Aminotransferase (AST)	472	10 – 34	U/L
Total Bilirubin	3.29	0.2 – 1.3	mg/dL
Direct Bilirubin	2.7	0 – 0.4	mg/dL
Lactate Dehydrogenase (LDH)	694	105 – 233	U/L
Lipase	348	0 – 160	U/L
Amylase	587	40 – 140	U/L

Clinical course

**Figure 1:**
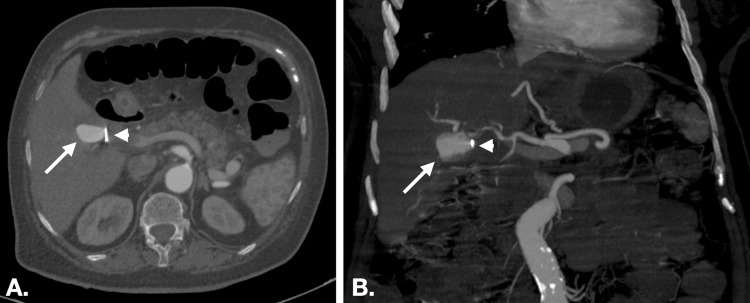
CT image showing the hepatic pseudoaneurysm adjacent to the right hepatic artery, with the larger arrow pointing to the pseudoaneurysm and the smaller arrow indicating the vascular metallic clip (from previous laparoscopic cholecystectomy) positioned tangentially to the PA. (A) Axial view. (B) Coronal view. PA: Pseudoaneurysm

During her hospitalization, she experienced a severe episode of epigastric pain accompanied by frank red blood in the nasogastric (NG) tube collection bag. An emergent upper GI endoscopy found the presence of frank blood emanating from the papilla of Vater. Further investigation via angio-CT identified a hepatic PA measuring approximately 28/17 mm in the hepatic hilum adjacent to the right hepatic artery, as depicted in Figure 1. Notably, a vascular clip from the previous cholecystectomy was found tangent to the PA, alongside a reduced quantity of hemoperitoneum.

Given the strong suspicion of hepatic artery pseudoaneurysm as the source of hemobilia and obstructive jaundice, an urgent surgical consultation was sought. The surgical team recommended interventional radiology evaluation, which led to a decision to proceed with stent placement.

Intervention

**Figure 2:**
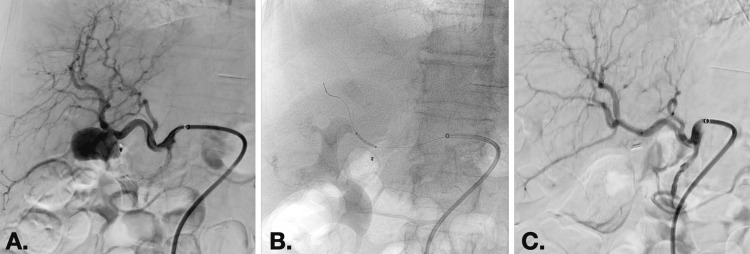
(A) Pre-intervention imaging showing the placement of the 6F guiding catheter within the celiac trunk. (B) Intraoperative image illustrating the navigation of the Graftmaster stent graft over the guidewire toward the pseudoaneurysm. (C) Post-intervention imaging demonstrating the successful obliteration of the pseudoaneurysm following the deployment of the stent graft.

An endovascular procedure under local anesthesia was carried out by the interventional radiology department. The PA, located in Segment V of the liver, had dimensions of approximately 38/26 mm and was fed by the right hepatic artery. Access was gained via a right common femoral artery approach. A 6F guiding catheter was placed in the celiac trunk (Figure 2A). Following this, a 4.5x19mm Graftmaster stent-graft (Abbott Laboratories, Chicago, Illinois, USA) was navigated over a 0.014” guidewire (Figure 2B). The stent graft was then deployed, resulting in the immediate obliteration of the PA (Figure 2C).

Post-intervention outcome

Post-procedure, the patient showed a favorable clinical course with the cessation of upper GI bleeding, remission of epigastric pain, and improvement in hematological parameters, though cholestasis persisted. She was discharged eight days post-stenting. At one-month follow-up, abdominal ultrasound showed a CBD diameter of 6 mm, and laboratory results were largely normalized, including a hemoglobin level of 11.4 g/dl.

## Discussion

Hepatic artery PAs represent a rare but potentially life-threatening complication often associated with LC, vascular injury, and iatrogenic trauma [14]. In our patient, the PA was located on the right branch of the hepatic artery, in the V-th hepatic segment. The modality employed for treatment was endovascular stent-graft placement, differing from the traditionally favored method of transarterial embolization (TAE). This alternative approach proved to be successful and offers valuable insights into the management of hepatic artery PAs.

Incidence and etiopathogenesis

LC, although minimally invasive, is associated with a documented increase in the frequency of both vascular and biliary injuries. Vascular complications associated with LC, such as pseudoaneurysm have been cited to occur at a rate of approximately 0.8% [16,17]. The pathogenesis is multifactorial and is not yet completely understood; however, vascular erosion secondary to clip encroachment, thermal or mechanical injuries during the surgical procedure, and continuous inflammation due to adjacent infected bile or collection, appear to be primary contributors [15].

Clinical presentation and diagnosis

The presentation of hepatic artery PAs is often insidious and can range from gastrointestinal bleeding to “Quincke's triad” consisting of right upper quadrant pain, jaundice, and hemobilia. However, it is essential to note that the triad is present collectively only in a minority of patients [18]. Timely diagnosis is crucial to managing this condition effectively. Our patient was evaluated using IV contrast computed tomography and later with computed tomography angiography. Selective hepatic, celiac, and superior mesenteric artery angiography plays a vital role in the real-time identification of pseudoaneurysms and possibly other causes of upper gastrointestinal bleeding following LC [19,20].

Treatment modalities

While TAE has traditionally been the standard intervention for PAs [20], contemporary expert consensus leans toward endovascular repair with stent grafts, particularly for aneurysms located in the proper hepatic artery [21]. This shift is attributed to the high efficacy of percutaneous treatments and the increasing acknowledgment that most hepatic artery aneurysms are amenable to non-surgical management.

For our patient, the anatomical characteristics of the hepatic artery PA, notably the broad neck of the lesion, called for a different therapeutic approach. Consequently, endovascular stent-graft placement was selected, an effective but less frequently employed modality for such cases. The intervention entailed selective catheterization of the right hepatic artery supplying the pseudoaneurysm, followed by the placement of a stent/stent graft. PAs with a neck width surpassing 4 mm present considerable challenges for conventional coil embolization, including risks of coil displacement and partial occlusion. These complications could result in persistent blood flow into the aneurysm and a heightened risk of rupture [21,22]. Our patient's PA had a neck sufficiently wide to pose these risks, influencing our decision in favor of stent-graft placement. This technique ensures a more dependable and secure exclusion of the pseudoaneurysm from vascular circulation, thus diminishing reperfusion likelihood.

When deliberating between TAE and endovascular stent-graft placement, several factors come into play. Anatomical suitability is paramount, requiring a thorough evaluation of the pseudoaneurysm's location, size, and neck morphology; hemodynamic stability is sought to decrease risks related to embolic agents, such as coil displacement or unintentional closure of adjacent vessels; the possibility of reperfusion, noted in literature as a complication in up to 20-25% of TAE procedures [14], is lessened with the stent-graft's reinforced arterial wall support; and lastly, therapeutic versatility. Unlike TAE, stent-grafting does not preclude subsequent TAE, offering a broader range of treatment options.

Prognostic factors and patient monitoring

The risk of rupture is substantially elevated for aneurysms larger than 5 cm, with reports indicating a rupture in 21%-80% of cases, associated with a mortality rate of up to 43%. [14,23]. Therefore, vigilant post-operative monitoring and early intervention are pivotal for optimal patient outcomes.

## Conclusions

Hepatic artery PAs are an uncommon but severe complication often related to LC among other causes. Our case study highlights the significance of early diagnosis through imaging techniques like IV-contrast CT. Additionally, it also demonstrates the viability of stent-graft placement when traditional TAE is unsuitable due to the PA's anatomical challenges. However, additional studies are essential to thoroughly assess the safety and long-term outcomes of stent-graft insertion compared to conventional TAE techniques.
